# Chitin Analysis in Insect‐Based Feed Ingredients and Mixed Feed: Development of a Cost‐Effective and Practical Method

**DOI:** 10.1111/jpn.14098

**Published:** 2025-01-16

**Authors:** Patrick Sudwischer, Björn Krüger, Werner Sitzmann, Michael Hellwig

**Affiliations:** ^1^ Research Institute of Feed Technology of IFF Braunschweig Germany; ^2^ Chair of Special Food Chemistry Technische Universität Dresden Dresden Germany; ^3^ C. Gerhardt GmbH & Co. KG Königswinter Germany; ^4^ Institute of Solids Process Engineering and Particle Technology Technische Universität Hamburg Hamburg Germany; ^5^ Amandus Kahl Holding GmbH Reinbek Germany

**Keywords:** chitin, compound feed, crude fibre, crude protein, insects, Kjeldahl, polymer, protein meal

## Abstract

Insects are used as an alternative sustainable, protein‐rich ingredient in fish, pet, pig and poultry diets. The significant difference between insect meals and common protein sources is the content of chitin. The nitrogen contained in chitin, which makes up 6.89% of the chitin mass, is detected as crude protein in the analysis and, therefore, deludes the crude protein content in a higher range. In this work, we developed a chitin analysis method that does not require expensive and specialized equipment within insect production and processing industries. The method is based on classical chemical methods such as crude fibre and nitrogen content, making it easily implementable within existing feed analysis. In the process of method validation, a recovery rate of over 95% for chitin in the presence of protein and a standard deviation of < 5% at concentrations as low as 2% was determined. Furthermore, determining chitin at a higher standard deviation of > 10% at concentrations as low as 2% is possible. The method was used to determine the chitin content in various products derived from insect breeding and processing. The chitin content was determined in four insect species (*Hermetia Illucens*; *Tenebrio molitor*; *Acheta domesticus*; *Bombyx mori*) and different developmental stages of the yellow mealworm (*T. molitor*), including larvae, pupae and beetles, as well as in commercial pet food. These results also allow for an estimation of the insect protein content, provided that the raw material is known.

**Abbreviations**
BSFL: black soldier fly larvae (*Hermetia illucens*)
DM: dry matter
LOQ: limit of quantification
TM: yellow meal worm (*Tenebrio molitor*)

## 1 Introduction

**Figure 1:**
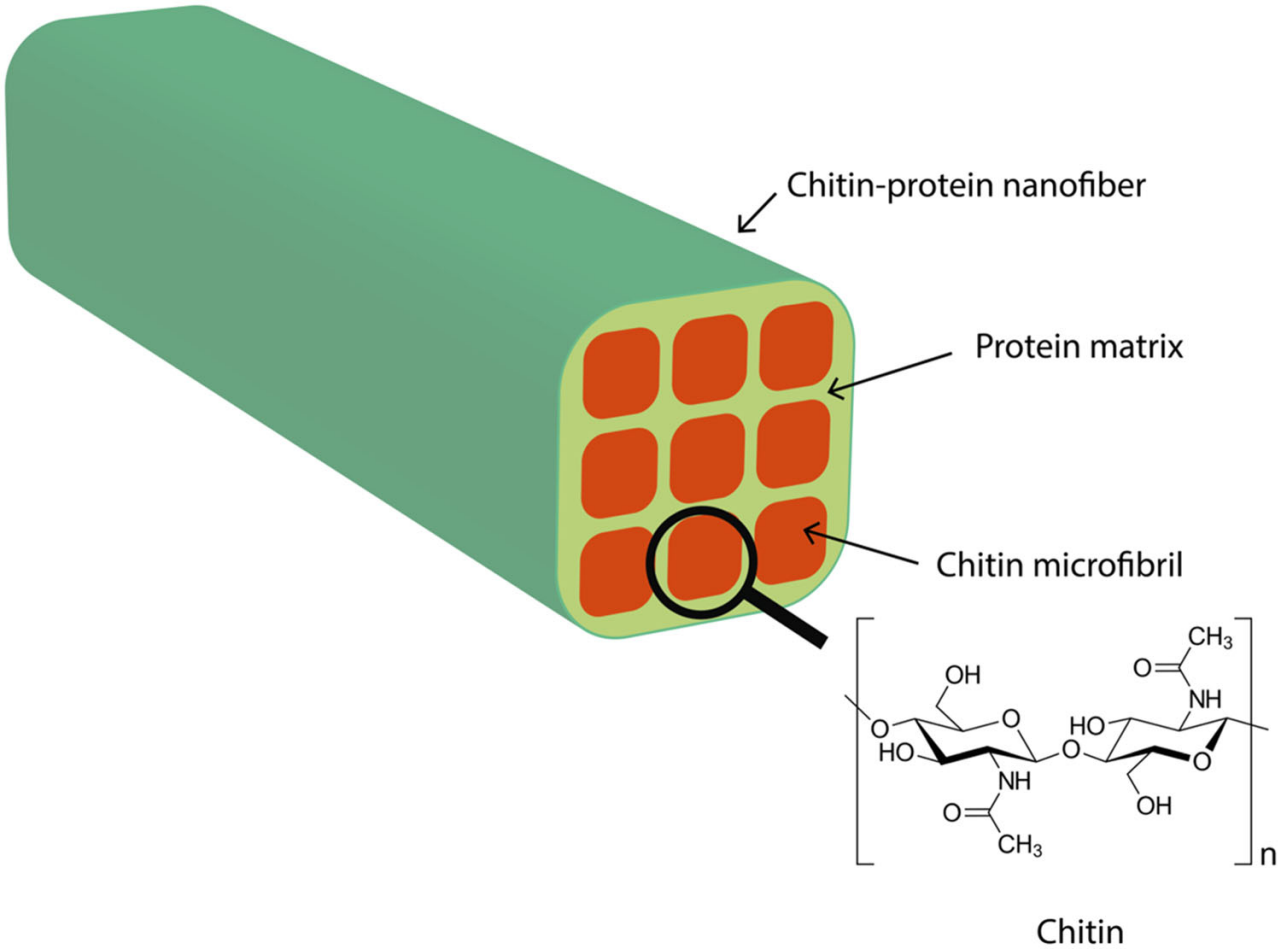
Chitin structure and occurrence in insect cuticle. [Color figure can be viewed at wileyonlinelibrary.com]

Compound feed production for livestock and pets worldwide accounts for approximately 1 billion tons each year. The European Parliamentary Research Service (EPRS) mentions 150.2 million tons compound feed in Europe and 24 million tons compound feed production in Germany per year (EPRS 2023). In Germany, 38% of this amount is fed to pigs, 28% to poultry, 28% to cattle for meat and egg production and 6% to pets and other animals (BLE Federal Office for Agriculture and Food 2023). To ensure the future protein supply for livestock and pets, insects are a valuable source of high‐quality protein. Besides providing essential nutrients, insects also offer various bioactive compounds that differ from plant‐based protein sources. Compounds such as catechols and chitin can have effects on animal health. Chitin, the second most abundant biopolymer in nature after cellulose, is a component of insects' exoskeletons. Mammals lack chitin, and therefore, it can be targeted by their immune system when they come into contact with the substance after ingestion. Janssen et al. (2017) published a correction of the nitrogen‐to‐protein conversion factor in insect‐based feedstuff. This N‐to‐P factor works only in pure insect feed material, but it is possible to calculate the accurate protein content for livestock diets. On the other hand, the global protein market calculates with the N‐to‐P factor of 6.25. In vivo trials conducted on fish, poultry and pigs have shown that chitin consumption affects the immune system (Van Huis and Gasco 2023; Hahn et al. 2020). In nature, chitin rarely exists in pure form. Usually, it is bound in a complex protein matrix with different compounds (Figure 1). As an example, the cuticle of crustaceans such as crabs and shrimp is composed of a matrix of protein and minerals (such as calcium) with nanofibrillar chitin, which provides flexibility and elasticity (Johnson and Peniston 1982; No, Meyers, and Lee 1989; Merzendorfer and Zimoch 2003). The cuticle of insects is composed of chitin in a matrix with cuticular proteins, lipids and other compounds (Kramer, Hopkins, and Schaefer 1995; Minke and Blackwell 1978; Moussian 2010; Mushi, Utsel, and Berglund 2014). Most insects contain only insignificant amounts of minerals in their cuticle, while some species, such as the pupae of the face fly (*Musca autumnalis*) and black soldier fly larvae (BSFL, *Hermetia illucens*), contain a significant amount of minerals in their cuticle (Dashefsky et al. 1976; Makkar et al. 2014). As an example, Makkar et al. (2014) published calcium and phosphorus contents of 7.56 g/100 g DM and 0.90 g/100 g DM, respectively, in BSFL. Chitin is a linear heteropolymer that consists of β‐(1→4)‐linked N‐acetyl‐d‐glucosamine units and a minor content of d‐glucosamine units. Chitin can be deacetylated through various chemical methods and enzymes. A commonly employed method is treatment with alkali, such as sodium hydroxide. This process results in a deacetylation rate of over 50%, leading to the production of chitosan (Hahn et al. 2020). Chitosan is also prepared from chitin on an industrial scale. The polymeric structure of chitin closely resembles that of cellulose, a linear β‐(1→4)‐d‐glucopyranose polymer (Finke 2007; Xiong et al. 2023). Chemically, chitin exhibits properties similar to cellulose and other β‐glucans. Generally, chitin is insoluble in water and undergoes random hydrolysis under low pH conditions (pH 1), while the polymeric binding remains stable under alkaline conditions. Alkaline deacetylation is possible but will not degrade the polymeric structure. Under hydrolysis conditions (e.g., 6 M hydrochloric acid [HCl] for 4 h at 110°C (Smets and Van Der Borght 2021), the chitin monomer and oligomer units react in different side reactions to form highly complex pigmented polymers. After hydrolysis into its monomeric units, these properties make the analysis of chitin a challenge for a complete detection and determination of the monomers (Kobayashi et al. 2021; Nitschke et al. 2011).

Chitin contains approximately 6.89% nitrogen (de Alvarenga 2011). This nitrogen is converted to ammonia during Kjeldahl digestion and, therefore, is part of the ‘crude protein’ parameter during Weende analysis of insect‐based feedstuff. This overestimation of crude protein due to co‐digestion of chitin contrasts with the principles of needs‐oriented animal nutrition, thus necessitating quantitation of both protein and chitin for accurate animal nutrition. Different methods are described for the determination of chitin. Finke (2007) reported a method to determine chitin in raw whole insects based on the crude fibre analysis, including various varieties of acid detergent fibre and neutral detergent fibre. However, this method cannot be applied to complex matrices including different fibre sources, such as compound feed. Another disadvantage of the method is that all crude fibre parameters are required to calculate the chitin content. The other way to determine chitin is the detection of the monomer units. In these methods the released monomers N‐acetyl‐glucosamine and glucosamine are quantified after total acid hydrolysis. The monomers can be detected using various chromatographic methods with subsequent colorimetric detection, refractive index detection or mass spectrometry (Smets and Van Der Borght 2021; Chen and Johnson 1983a, 1983b). All methods that use a total hydrolysis have several disadvantages. The conditions of acid hydrolysis enable many cross‐reactions between amino groups of amino acids, proteins and chitin. Total hydrolysis of polysaccharides generates a range of different new pigmented polymers. Finally, a preferentially high degree of hydrolysis must be reconciled with a preferentially low occurrence of by‐products. In a recent work, chitin was quantified in edible mushrooms (Nitschke et al. 2011). With this method, chitin is converted to chitosan under strongly alkaline (saturated potassium hydroxide solution) conditions and subsequent detection by a colour reaction between chitosan and polyiodide (1% Lugol solution) in the solid state on a thin layer chromatography plate. The big advantage of this method is that chitosan is detected directly without hydrolysis to monomeric units. Proteins are removed by subjecting the material to alkaline treatment, predominantly employing a solution of diluted sodium hydroxide. This deproteination procedure has a dual effect of extracting some of the dyes and soluble lipids found in the exoskeleton, all the while maintaining the integrity of the chitin polymer structure. This preservation of the polymer structure is evident, for instance, in the isolation process from crab exoskeletons and BSFL exuviae (Xiong et al. 2023; Hahn et al. 2020). On the other hand, sample preparation is somewhat complex for an industrial monitoring analysis, and the parameters for deacetylation have to be carefully chosen and controlled. A problem is the effect of an incomplete deacetylation, which results in a partial solubility and in an underestimation of chitin. The correlation between deacetylation rate and colour intensity has not been investigated yet.

The objective of this study is to assess the chitin content through a method that is easily manageable, avoiding the need for expensive or specialized equipment. The intention is to demonstrate that the classical Weende analysis method can be adapted to incorporate chitin parameters effectively. Through this, a detailed description of feed materials derived from insects using the Weende analysis method should be achieved. Furthermore, the study aims to tackle the quality control challenges associated with insect production. It will delve into the obstacles, proposing solutions for chitin analysis through the modification of a traditional chemical analysis method.

## 2 Materials and Methods

### 2.1 Chemicals

The following chemicals were purchased from commercial suppliers: petroleum ether 40°C–60°C, acetone (VWR, Darmstadt, Germany) Kjelcat Cu (5 g K_2_SO_4_ + 0.5 g CuSO_4_·5H_2_O) (C. Gerhardt Analytical Systems, Königswinter, Germany), sulphuric acid solution 0.05 M, boric acid > 98% p.a., sulphuric acid 96% (m/m), hydrogen peroxide solution 35% (m/m), chitin from crab shell (M = 400,000 g/mol), sodium hydroxide (Carl Roth, Karlsruhe, Germany).

### 2.2 Samples

Various patterns of samples (shown in Tables 1 and 2) were employed in this study during the development of the presented method. These included determination of recovery rate and hydrolysis kinetics, determination of repeatability and reproducibility, as well as the application of the method on commercial samples. For the creation of a recovery sample, soybean meal was chosen as the protein component to form the basis of the matrix. Pure chitin was mixed in, which needs to be recovered from this matrix to test the efficiency and practicability of the method. Furthermore, incomplete hydrolysis of the protein would cause a significant error in the method and increase the recovery rate. To examine this effect, we created a standardized mixture of soybean meal and chitin for the method recovery rate. To determine the recovery rate of the method, soy meal was spiked with pure chitin from crab shell (Table 1). Soy meal and chitin were prepared by grinding to a size below 0.5 mm (milling sieve 0.5 mm; Retsch ZM‐1). Subsequently, a stock mixture with a chitin content of 9.22 g chitin/100 g DM was prepared from these ground products. Recovery samples were then generated by mixing the stock mixture, which contained 9.22% chitin in dry matter (DM), with pure chitin and soy meal (Total nitrogen = 6.18 g nitrogen/100 g) at three concentrations. To create these samples, the stock mixture was diluted with soy meal to achieve concentrations of 4.61, 2.30 and 1.15 g chitin/100 g. For example, for the sample containing 4.61 g chitin g/100 g, 100 g of the stock mixture was mixed with 100 g of the soy meal. All samples were mixed in a 5‐L batch mixer (Lödige, Paderborn, Germany) for 5 min. As a control sample, pure soy meal without chitin was used. Since soy meal does not contain polysaccharide‐bound nitrogen, it is suitable as a control group to verify the completeness of the hydrolysis. The positive control was conducted with pure chitin to ensure that no significant losses occurred during the processing. As a blank sample, the method was run without sample material. Furthermore, the study of the time course of hydrolysis also used insect rearing by‐products, which consist of a mixture of frass, exuviae and dead insects. This sample was used solely in the hydrolysis study as a stress test for a heterogeneous sample matrix to evaluate the effectiveness of the hydrolysis conditions.

**Table 1:** Composition of samples for the determination of the recovery rate of chitin.

Sample	Chitin concentration [g/100 g]	Mass pure chitin [g]	Mass pure soy meal [g]
Soy meal	—	0.00	100.00
Recovery 1	1.15	1.25	98.75
Recovery 2	2.30	2.50	97.50
Recovery 3	4.61	5.00	95.00
Recovery 4	9.22	10.00	90.00
Pure chitin	92.25	100.00	0.00

**Table 2:** Commercial sample list and declared nutrient value^a^.

Sample	Insect content	Crude fat	Crude fibre	Crude protein (6.25)	DM^b^	Ash
BSFL dry B^c^	Pure	36.0	9.0	42.0	95.0	6.0
TM dry^c^	Pure	34.9	5.4	48.6	94.6	3.0
Crickets dry	Pure	31.6	6.5	54.8	93.8	3.5
Silkworm dry	Pure	30.0	4.5	53.0	92.0	5.0
*Zophobas morio*	pure	13.0	n.s.	51.3	39.0	1.0
BSFL protein meal A	Pure	8.8	10.0	52.0	97.0	10.0
BSFL protein meal B^c^	Pure	11.7	n.s.	60.0	95.8	n.s.
BSFL protein meal C	Pure	10.1	8.9	53.6	95.0	12.8
TM protein meal^c^	Pure	5.1	8.4	69.5	95.3	6.9
Dog wet food A	5%	21.2	7.8	20.4	24.5	0.8
Dog wet food B	5%	21.6	7.9	20.7	24.1	0.8
Cat wet food	32%	19.6	1.4	50.0	28.0	6.4
Dog dry food C	15%	10.0	3.4	21.1	95.4	6.5
Dog dry food D	10%	12.0	2.7	22.0	92.3	7.1
Cat dry food	14%	16.5	4.0	33.7	91.5	8.2
TM larvae	Pure	n.s.	n.s.	n.s.	n.s.	n.s.
TP puppet	Pure	n.s.	n.s.	n.s.	n.s.	n.s.
TM larva skin	Pure	n.s.	n.s.	n.s.	n.s.	n.s.
TM beetle	Pure	n.s.	n.s.	n.s.	n.s.	n.s.

*Note:* n.s., value is not specified.

^a^ All data are given in g/100 g DM. All details are taken from the product label declaration.

^b^ DM was determined as described in the Materials and Methods section.

^c^ Larvae are raw material of protein meal.

To assess the repeatability and reproducibility of the method, we used recovery samples numbered 2–4 (Table 1), as well as TM dry and TM protein meal (Table 2). These samples were subjected to the complete analytical workflow described below.

To test the method under realistic conditions, 19 samples were obtained from commercial suppliers. These samples covered representatives of all technologically relevant types of insect products, including five whole‐insect products, four protein meals, six pet mixed feed products (wet and dry) and four TM farming products (Table 2).

### 2.3 Preparation of Samples for Analysis

For quantitation of chitin in fresh insects or moist feed samples, the samples were dried for 4 h at 103°C (Reg. (EU) 152/2009). The dry sample was ground by a laboratory mill (milling sieve 1.0 mm; Retsch ZM‐1) below a particle size of 1.0 mm. Samples with a fat content of up to 10% were degreased using petroleum ether. To do this, the samples were overlaid with ether and left to settle for 10 min. The ether layer was removed and the remaining solvent evaporated at room temperature under a fume hood.

### 2.4 Deproteination by Alkaline Hydrolysis

**Figure 2:**
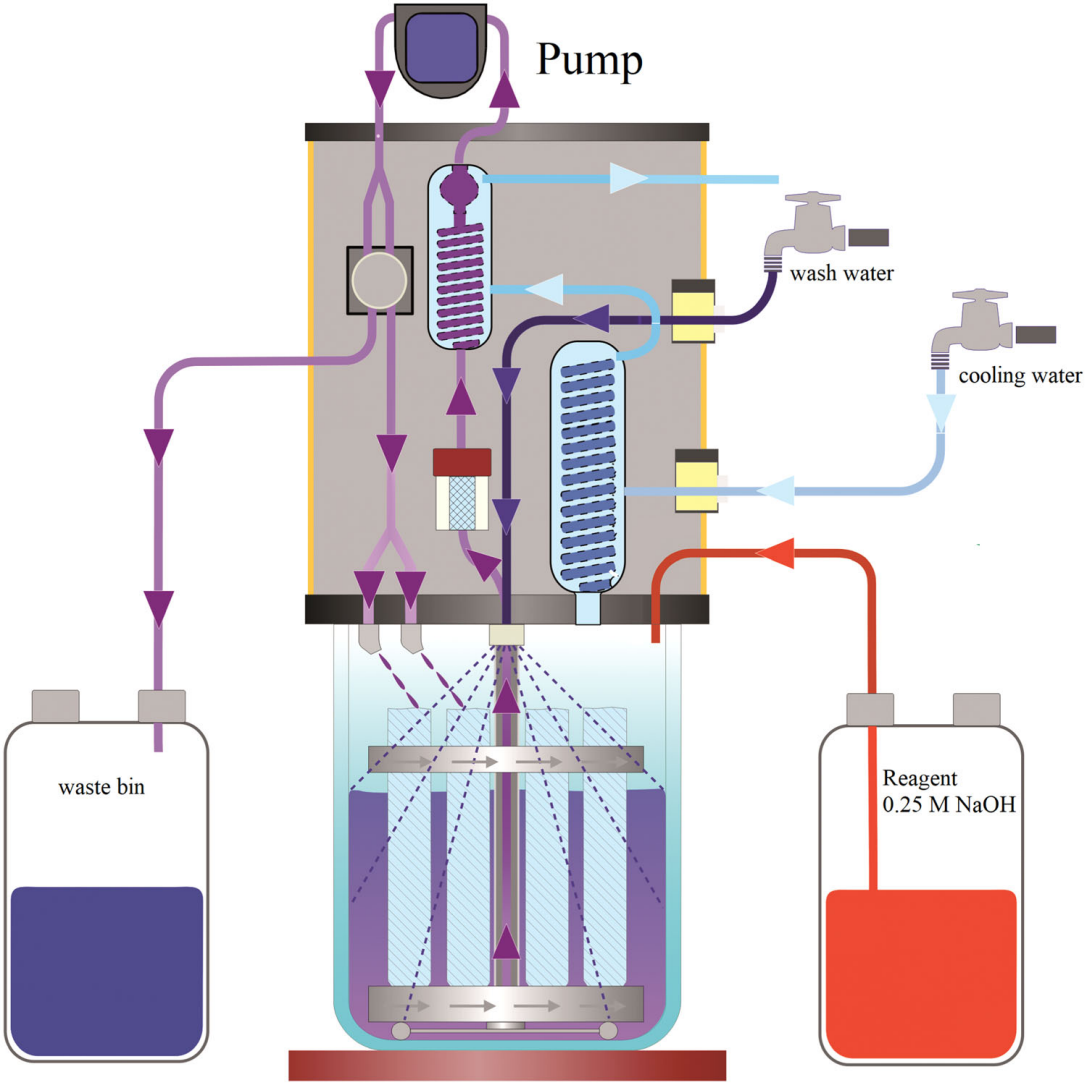
Functional diagram of the Fibretherm system. [Color figure can be viewed at wileyonlinelibrary.com]

Deproteination was performed with a Fibretherm analyzer (Figure 2; C. Gerhardt Analytical Systems) using a FibreBag S sieve mesh. The FibreBag technique involves performing digestion and filtration within a sufficiently sized filter bag composed of a high‐precision specialized textile (Supporting Information S1: Figure S1). This textile material ensures consistent and replicable filtration circumstances. After the alkaline digestion of protein, the used FibreBags are completely dissolved into carbon dioxide and water along with the remaining sample as part of the nitrogen determination. This ensures that each analysis is conducted under consistent and standardized filtration conditions, promoting consistently reproducible results.

Samples of 1 g were boiled for 60 min in 1250 mL of 0.25 M NaOH solution per 12 samples. After hydrolysis, all samples in a Fibretherm‐batch were washed twice with 1250 mL hot water for 5 min each. Following the washing process, the FibreBags were dried for 4 h at 103°C.

### 2.5 Time Course of Hydrolysis

The time‐course experiment utilized a 1 g sample of soy meal, soy meal spiked with chitin and an insect rearing byproduct. The insect rearing byproduct comprised frass, feeding substrate, dead insects and exuviae. This byproduct was included in the experiment to serve as a stress test for determining the endpoint of deproteination.

Soy meal without spiked chitin served as the control sample for recovery experiments. Soy meal with chitin (4.2 chitin g/100 g DM) was used as a positive sample. Prior hydrolysis, the sample was spiked with a chitin standard.

The experimental procedure involved collecting samples after 0, 15, 30, 45, 60, 75 and 90 min of reaction time. Duplicate determinations were performed for each time point. After each time point, the reactions were stopped by washing the samples with deionized water. The nonhydrolysed nitrogen content in the residue in the FibreBags was subsequently determined using the Kjeldahl method.

### 2.6 Nitrogen Determination

Nitrogen determination in the residue after alkaline hydrolysis was performed using the classical Kjeldahl method for nitrogen analysis. Acid digestion took place in a Kjeldahl flask by adding two Kjelcat tablets, 0.5 mL of hydrogen peroxide solution (35%; m/m), and the dried FibreBag from alkaline deproteination. Subsequently, 20 mL of sulphuric acid (96%; m/m) was added to the sample. The digestion process occurred in a Turbotherm digestion unit (C. Gerhardt Analytical Systems, Germany) for 175 min. Upon completion of digestion, the sample vials were allowed to cool for 15 min. Following this, 100 mL of water was added and thoroughly mixed.

Steam distillation was performed using a Vapodest 20S (C. Gerhardt Analytical Systems, Germany). The distillation process started by adding 85 mL of sodium hydroxide solution (40%; m/m) and allowing the distillation to proceed for 5 min. In a separate receiving vessel, 80 mL of boric acid solution (20 g/L) was added for collection of the steam. Subsequently, titration was performed using 0.05 M sulphuric acid with pH control until pH 5.2 had been reached. A blank sample was included to determine any nitrogen from sources other than the feed samples. For this blank sample, all steps of analysis (digestion, distillation, titration) were included, but no substance was weighed into the Kjeldahl flask.

The chitin content was calculated from the nitrogen content of the samples using the following formula.

$$\mathrm{Chitin}=\frac{\left(V-V_{\mathrm{blank}}\right)\;\cdot \;c_{\mathrm{eq}}\;\cdot \;1.4007}{m_{\mathrm{sample}}\;\cdot \;14.007}\;\cdot \;203.19.$$

V, Volume of 0.05 M sulphuric acid used for titration of the sample [mL]; V_blank_, Volume of 0.05 M sulphuric acid used for titration of the blank test [mL]; c_eq,_ Equivalent concentration of H^+^ in the standard solution [mol/l]; 1.4007, Factor converting the titrated volume (in mL 0.1 mol/l H^+^) into the mass of nitrogen (in mg); 14.007, Molar mass of nitrogen; m_sample_, Weight of the sample [g]; 203.19, Molar mass of a chitin monomer.

The first term in this formula represents the amount of substance in mol/100 g. This quantity is equivalent to the amount of chitin monomers (N‐Acetylglucosamine) since each monomer contains a single nitrogen atom. Therefore, the total amount of chitin can be calculated from the molar concentration using the molar mass of the monomer. The molar mass of N‐acetylglucosamine is reduced by 18.02 because of the loss of one molecule of water during the formation of a glycosidic bond in the chitin polymer.

### 2.7 Infrared Spectroscopy

To confirm that the residue in the Fibrebag after deproteination is indeed isolated chitin (and other polysaccharides) and not another material, an ATR‐IR spectrum of both the residue and the pure substance was recorded using a Bruker alpha FTIR spectrometer equipped with a ZnSe crystal detector device. Each spectrum was obtained by collecting 24 scans with a resolution of 2 cm⁻¹. The spectral range covered in the analysis was from 370 to 7500 cm⁻¹. The ATR data analysis was performed using the OPUS Viewer software version 6.5 (Bruker, Germany).

### 2.8 Method Validation

For method validation, we followed the guidelines provided by the German Federal Environment Agency (Wellmitz and Gluschke 2005). All formulas utilized are detailed in the supplement. Our assessment focused on the repeatability of the measuring instrument (blank value), as well as the repeatability (referring to the same procedure conducted on identical samples within the same laboratory, by the same operator, using the same equipment, at short time intervals), and the reproducibility (indicating that the same procedure was applied to identical samples in different laboratories by various operators with similar equipment setups) between IFF (Braunschweig, Germany) and C. Gerhardt Analytical Systems GmbH (Königswinter, Germany), using equal samples of dried TM and TM insect meal press cake (Table 2). To ensure robustness, the method was applied to 312 measurements across 33 batches of these samples (see Tables 1 and 2).

### 2.9 Statistical Analysis

The statistical analysis involved calculating the minimum and maximum results, as well as determining the standard deviation (SD) and relative standard deviation (RSD) using Excel. To determine the hydrolysis endpoint, a one‐factor analysis of variance (with a significance level of *p* = 0.05) is conducted using the hydrolysis time course results from 45 min up to 90 min. Precision indicates how much the analytical values scatter due to random errors. Statistically, precision is described by the SD or confidence interval (±5%). It is distinguished between:

#### 2.9.1 Repeatability of Measuring Instruments

Random errors (without samples) in the total analytical system are caused by the instrument itself (IFF and C. Gerhardt).

#### 2.9.2 Repeatability

Precision under repeatability conditions (Repeatability SD); measure of repeatability (i.e., the same procedure on the identical test object in the same laboratory by the same operator with the same equipment at short time intervals). This is calculated by the mean value of the SD of all measurements in the IFF.

#### 2.9.3 Reproducibility

Precision under reproducibility conditions (Reproducibility SD); measure of comparability or transferability (i.e., the same procedure on the identical test object in different laboratories by different operators with different equipment, for example, in the context of a proficiency test). This is calculated by the SD between batches of all measurements in the IFF and C. Gerhardt Analytical Systems. If not otherwise stated, data assessment was also performed using Excel.

## 3 Results

### 3.1 Time Course of the Deproteination Reaction

**Figure 3:**
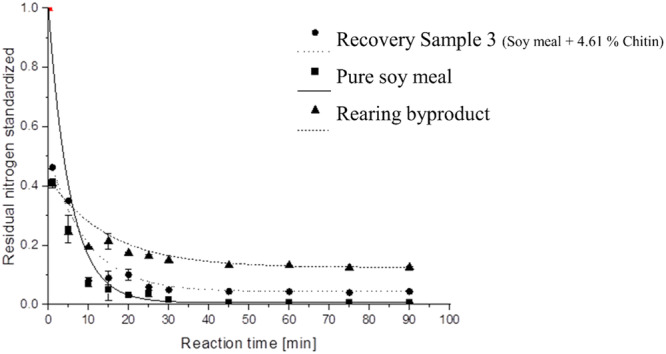
Insoluble residual nitrogen during different incubation times at 100°C in the presence of 0.25 M NaOH for deproteination, standardized to 100% of initial nitrogen content.

Polymer‐bound nitrogen withstanding alkaline hydrolysis is defined as chitin‐bound nitrogen in the present work. For the elucidation of the chemistry underlying the process of chitin isolation, it is necessary to consider the time course of protein hydrolysis to define the endpoint of the deproteination step. The fraction of nonhydrolysed nitrogen in the samples is shown in Figure 3 (Supporting Information S1: Table S1). Furthermore, the identity of the isolated chitin was confirmed through ATR‐IR Spectroscopy. This analysis helped verify that the residue following alkaline hydrolysis closely resembled pure chitin (or other polysaccharides such as cellulose) and no other significant nitrogen sources. (Supporting Information S1: Table S2 and Figure S2).

### 3.2 Method Validation Parameters

**Figure 4:**
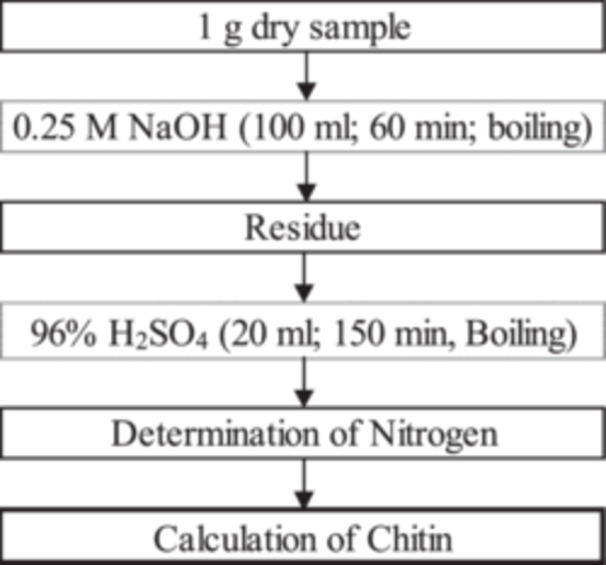
Analytical workflow for chitin determination.

Based on the results of deproteination and the isolation of polysaccharides/chitin in the sample, a workflow was created for an easy chitin analysis method that does not require specific analytical equipment (Figure 4). The recovery rate was tested by applying the method to six different chitin concentrations, ranging from pure chitin to four mixtures of chitin with soy meal and with pure soy meal serving as a chitin‐free sample. This aimed to simulate various chitin concentrations in a sample containing a protein matrix. The results for the determination of chitin recovery are shown in Table 3.

**Table 3:** Recovery rate of chitin in different concentrations.

Chitin content^a^	*n*	Min‐max^a^	Mean ± S.D.^a^	Recovery [%]	Nitrogen content ± S.D.^a^
0.00	15	0.49–0.99^b^	0.82 ± 0.13^b^	< LOQ	0.07 ± 0.02
1.15	8	1.49–1.68	1.59 ± 0.18	138.3 ± 0.2	0.09 ± 0.05
2.30	12	2.32–2.66	2.48 ± 0.42	107.8 ± 0.2	0.17 ± 0.01
4.61	22	4.27–4.87	4.56 ± 0.15	98.9 ± 0.1	0.32 ± 0.04
9.22	12	8.63–9.53	9.15 ± 0.21	99.3 ± 0.1	0.63 ± 0.01
92.25	27	88.79–93.00	90.67 ± 1.01	98.3 ± 0.1	6.29 ± 0.13

^a^ Contents are given in g/100 g.

^b^ Nitrogen content is below limit of quantification (LOQ).

The results for the determination of the repeatability including SD and RSD of various samples are shown in Table 4. The repeatability of measuring instruments, and the reproducibility of the method (relative variation between samples which are measured by IFF and Gerhardt laboratories), are presented in Table 5.

**Table 4:** Repeatability of the chitin determination in different sample matrices is described with the standard deviation (SD).

Sample	*n*	Mean chitin content (g/100 g)	SD^a^ (g/100 g)	RSD (%)
TM dry	90	6.4	0.2	3.2
TM protein meal	118	9.7	0.1	1.4
Chitin	58	91.6	0.8	0.9
Recovery 4	12	9.1	0.2	2.3
Recovery 3	22	4.6	0.1	2.3
Recovery 2	12	2.6	0.1	3.9

**Table 5:** Method validation parameters.^a^

Parameter	Minimum	Average	Maximum
Repeatability of measuring instruments^b^	—	0.04	—
Repeatability^c^	0.05	0.27	2.04
Reproducibility^d^	0.34	1.36	2.23

^a^ All results are given in percent.

^b^ Random errors (without sample) in total analytical system are caused by the instrument itself (IFF and C. Gerhardt).

^c^ Random error of the analysis results. It is determined as the mean of the standard deviations overall measurements series in the same laboratory (IFF) (*n*_Measurement _= 128).

^d^ Measurement error between two labs (*n*_Measurement _= 312; IFF *n*_IFF_ = 128 and C. Gerhardt *n*_Gerhardt_ = 184).

**Figure 5:**
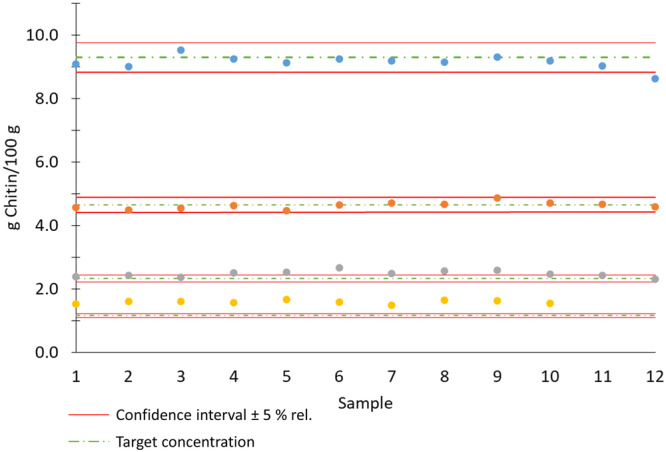
Individual measurements of different recovery samples (chitin spiked soy meal) with different chitin concentrations with a confidence interval of ±5%. [Color figure can be viewed at wileyonlinelibrary.com]

The confidence interval of the method is depicted in Figure 5, encompassing values from four measurement series with chitin concentrations of 9.22, 4.61, 2.30 and 1.15 g/100 g DM. The confidence interval was set at ±5% for chitin concentrations between 9.1 and 4.5 g/100 g DM and ±10% for chitin concentrations between 4.5 and 2.0 g/100 g DM of the expected target mean value.

### 3.3 Determination of Chitin in Different Insect and Insect‐Based Commercial Samples

To test the method under real conditions, chitin contents were determined in different commercial samples and TM farming products. The results are shown in Table 6.

**Table 6:** Chitin content of different insect/insect‐based samples.^a^

Sample	Chitin content (g/100 g DM)	SD^d^ (g/100 g DM)	RSD^e^ (%)
BSFL dry B^b^	7.3	0.3	4.1
TM dry^b^	6.3	0.3	4.8
Crickets dry	8.2	0.3	3.7
Silkworm dry	4.7	0.4	8.5
BSFL protein meal A^c^	13.6	0.2	1.5
BSFL protein meal B^b^	10.1	0.1	1.0
BSFL protein meal C	8.6	0.1	1.2
TM protein meal^b^	9.3	0.3	3.2
Dog wet food A	1.2	0.1	8.4
Dog wet food B	1.3	0.1	7.7
Cat wet food	4.4	0.2	4.6
Dog dry food C	1.8	0.6	33.4
Dog dry food D	2.2	0.5	22.7
Cat dry food	2.2	0.1	4.6
*Zophobas morio*	6.8	0.2	3.0
TM larvae	7.7	0.1	1.3
TM puppet	3.8	0.1	2.4
TM larvae skin^c^	42.8	0.5	1.2
TM beetle	34.0	0.4	1.2

^a^ All results are given in g/100 g DM. *n* = 5.

^b^ Larvae are raw material of protein meal.

^c^ *n* = 3 (Small sample mass).

^d^ SD = standard deviation.

^e^ RSD = relative standard deviation are given in percentage.

### 3.4 Influence on Crude Protein Content

The Influence of chitin‐bound nitrogen on the crude protein content is described in Table 7. To correct the crude protein content the chitin‐bound nitrogen is subtracted from the total nitrogen amount.

$$N_{\mathrm{corrected}}=N_{\mathrm{total}}-N_{\mathrm{Chitin}}.$$

**Table 7:** Influence of chitin content on crude protein of different commercial insect/insect‐based samples.^a^

Sample	Insect content	Chitin content	Chitin nitrogen	Total nitrogen	Crude protein (F6.25)^b^	Corrected protein	Relative overestimation [%]
Crickets dry	100	8.2 ± 0.3	0.6	8.8	54.8	51.0 ± 0.1	7.5 ± 0.1
BSFL protein meal B^b^	100	10.1 ± 0.1	0.7	9.6	60.0	55.3 ± 0.1	8.5 ± 0.1
BSFL protein meal C	100	8.6 ± 0.1	0.6	8.6	53.6	49.7 ± 0.1	7.8 ± 0.1
Cat wet food^c^	32	4.4 ± 0.2	0.3	8.0	50.0	48.1 ± 0.1	4.0 ± 0.1
Dog dry food C^c^	15	1.8 ± 0.6	0.1	3.7	21.1	20.3 ± 0.1	3.9 ± 0.1
Cat dry food^d^	14	2.2 ± 0.1	0.2	5.4	33.7	32.7 ± 0.1	3.1 ± 0.1
*Zophobas morio*	100	6.8 ± 0.2	0.5	8.2	51.3	48.4 ± 0.1	6.0 ± 0.1

^a^ All values are given in g/100 g DM sample.

^b^ Product declaration (F6.25 according to Regulation [EC] 152/2009).

^c^ No declaration of the insect species.

^d^ Insect species is BSFL.

The determination was conducted in accordance with Regulation (EC) No 152/2009 using a conversion factor of 6.25.

## 4 Discussion

### 4.1 Alkaline Deproteination and Analytical Principle

For the elucidation of the deproteination reaction, Figure 3 illustrates the progress of the reaction over time. The decreasing concentration of polymer‐bound nitrogen in the sample (Figure 3, Supporting Information S1: Table S1) demonstrates a typical reaction kinetic for a first‐order reaction. At a more detailed level, this reaction can be described as a depolymerization process that follows pseudo first‐order kinetics. This is due to the fact that the decrease in concentration of the abundant hydroxyl ion at pH 14 is considered negligible when using 1250 mL of 0.25 M NaOH for every twelve 1 g samples. The degradation rate of the residual nitrogen content follows the expected polymeric degradation function $\left[\mathrm{polymer}\right]={\left[\mathrm{polymer}\right]}_{0}e^{-\mathrm{kt}}$ (Figure 3). The residual nitrogen in the sample asymptotically approaches a minimum value. The residual amount of nitrogen after 45 min is statistically not significantly (*p *> 0.05) diverse from the amount after 60 min (Supporting Information S1: Figure S3). This minimum value is dependent on the specific sample property, especially for the chitin content which is stable under alkaline conditions. Chitin, a polysaccharide, is not degraded under these conditions unlike other nitrogen sources in the sample, primarily protein (Hahn et al. 2020, Xiong et al. 2023). The residual nitrogen content in the sample after alkaline deproteination was found to be directly proportional to the chitin content, as depicted in the hydrolysis time course shown in Figure 3 (Supporting Information S1: Table S1), and it is also evident from the recovery study (Table 3). To ensure that no additional nitrogen source was present alongside chitin, the reaction time was extended to 60 min. After this period a stable minimum of residual nitrogen content was observed for HP soy meal (0.11 g_Nitrogen_/100 g), HP soy meal spiked with 4.61% chitin (0.58 g_Nitrogen_/100 g), and insect material (1.30 g_Nitrogen_/100 g). There was no significant decrease in the residual nitrogen content after 60 min. After linearizing the determined degradation (Supporting Information S1: Figure S3) rate, it can be observed that the reaction reaches a stagnant state after 60 min at the latest.

This finding indicates that the rate of protein degradation slows down over time and eventually reaches a very low level. The decrease in the degradation rate suggests that after 60 min the protein degradation process becomes less significant, and additional degradation is unlikely to occur, most probably because the protein has been removed completely. This information is important for understanding the kinetics of the reaction and to set the optimal reaction time for accurate analysis. These results indicate that a reaction time longer than 60 min is not necessary for effective deproteination.

In the context of this study, chitin is defined as the nitrogen‐containing insoluble residue that remains after subjecting a feedstuff to alkaline treatment with 0.25 M NaOH at 100°C for 60 min.

It is important to note that in insect‐based feedstuff this nitrogen‐containing residue primarily consists of chitin (Finke 2007, Makkar et al. 2014). In addition to its definition as the nitrogen‐containing insoluble residue after alkaline treatment, it is important to acknowledge that chitin can coexist with other polysaccharides, such as cellulose, in certain samples. This presence of cellulose and other polysaccharides can potentially influence the determination of chitin when methods relying on crude fibre are applied. On the contrary, the analytical method employed in this study aims to selectively quantify chitin as nonalkaline hydrolysable nitrogen (Table 3). It is important to note that, serving as the basis for chitin measurement, the presence of other polysaccharides has no effect on the accuracy of nitrogen determination in our study. The method's specificity for chitin relies on the assumption that other polysaccharides do not interfere with the nitrogen determination after deproteination in the analytical workflow. The utilization of alkaline treatment and specific reaction conditions allows for the selective isolation of chitin from other nitrogen‐containing components in the feedstuff because the latter either have low molecular weights (e.g., amino acids, vitamins) or form polymers that are not stable in alkali (e.g., proteins). Thus, we assume that all nitrogen‐containing compounds except chitin are removed from the FibreBags during alkaline treatment either directly or after alkaline hydrolysis due to their low molecular weight. This provides confidence in the measurement of chitin content and ensures that the method captures the desired analytical target.

### 4.2 Usability of the Method

The developed method exhibits a high recovery rate (98.3%–107.8%, Table 3) for chitin within the range of 2.3–92.3 g chitin/100 g DM. The recovery rate increases in chitin concentrations below 2.3 g/100 g, reaching an overestimation of 138.3%. These overestimations below 2.3 g/100 g of chitin are attributed to the presence of background noise in the nitrogen determination, posing a limit of quantification for this method. In experiments with pure soy meal without chitin addition, a baseline nitrogen content of 0.07 g/100 g was determined. With an addition of 1.15% chitin, the nitrogen content rises to 0.09 g/100 g (Table 3). Here, a significant distortion of the analysis results can be observed, as the systematic errors of the analysis system become proportionally larger. Due to this difference between the measured value and the baseline noise, the limit of quantification is considered to be above this point. In comparison to other methods, such as those reported by Nitschke et al. (2011) with 101 ± 8% and Smets and van der Borght (2021) with 98.5%–105.8%, the recovery rates of our method demonstrate very good performance.

The method has been validated for a chitin range of 0.023–0.923 g of chitin per 1 g sample batch. It is hypothetically suggested that increasing the sample weight could potentially enhance the method's detection limit to below 0.023 g of chitin per 1 g of sample. It should be noted that using more than 2 g of sample can lead to foaming in the reactor vessel, resulting in potential sample loss. Chitin determination of a content between 1 and 2 g/100 g results in a higher relative SD of ±35% and requires an increase of the used sample mass from 1 to 2 g. The confidence interval (Figure 5) of the method needs to be adjusted from below ±5% to ±35% for chitin concentrations below 2 g/100 g DM.

In feed industry, when using insects as a protein source (cat feed up to approx. 30% insects; dog feed 10%–20% insects, as indicated on commercial feed samples), it is common for the chitin content to be above 2%. This indicates that the developed method for chitin analysis is applicable for insect‐based feed formulations. By accurately quantifying the chitin content in these feed products, it becomes possible to assess their nutritional composition precisely and ensure their suitability for specific animal diets. The ability to measure chitin content with precision and reliability using the developed method provides valuable insights for the evaluation and quality control of insect‐based feed ingredients. This information is crucial for formulating balanced diets and optimizing animal nutrition, especially in cases where insects serve as a significant protein source. Moreover, the method demonstrates excellent repeatability across all measurements, with a relative SD below 5% (*n* > 300). This indicates a high level of precision in the analytical results. The comparison precision analysis reveals the ability to transfer the method and its results to other laboratories, as the measured deviations generally fall within a confidence interval of ±5%.

Unlike other methods, such as the one proposed by Finke (2007), which calculates chitin content based on crude fibre parameters, the developed method allows for chitin quantification even in the presence of other polysaccharides. This enables the analysis of complex feed mixtures. In contrast to the method of Nitschke et al. (2011), which involves laborious chitin isolation and derivatization to chitosan, the method developed here offers a more streamlined and time‐efficient sample preparation process. The developed method allows for efficient sample preparation in a standard feed laboratory setting. Other methods which are reported in the literature, such as those described by Semt et al. (2021) or Nurfikari and de Boer (2021), utilize LC‐MS and exhibit comparable accuracy (95.2%–111.6%) and a relative SD overall samples between two different laboratories (IFF and Gerhardt) of < 3.5%. However, these methods require total hydrolysis using 6 M HCl, which can be challenging to perform correctly. If not properly executed, the excessive formation of by‐products during hydrolysis can significantly distort the analysis results. Furthermore, the limitations and challenges associated with total hydrolysis of chitin in complex matrices, such as feed mixtures, have not been specifically investigated in this study. Further research is needed to evaluate the effectiveness and reliability of total hydrolysis in accurately quantifying chitin content in complex feed matrices. Moreover, these methods often require expensive equipment, limiting their widespread implementation.

In contrast, the developed method can easily be implemented in a conventional feed laboratory, making it more accessible and cost‐effective for routine analysis. The method offers a reliable and practical approach for chitin analysis, allowing for precise quantification of chitin content in various feed samples, including those with complex matrices. Considering the recovery and hydrolysis experiments and the assumption that only chitin‐bound nitrogen is found after hydrolysis, this method enables feed producers, nutritionists and quality control personnel to acquire precise chitin content data. This supports informed decision‐making and guarantees the quality and safety of insect‐based feed ingredients. These findings emphasize the usability, robustness and reliability of the developed method for chitin analysis, providing valuable insights for its application in various settings.

In Table 6, we present the method parameters for various commercial samples and insect farming products. In the case of crickets, there are variations in the reported results: Finke (2007) reported a chitin content of 6.8%, while Hahn, in the chitin isolation process, described values ranging from 4.3% to 7.1%. Our method yielded a chitin content of 8.2%, which is generally higher compared to the other two studies, all of which employ an acidic hydrolysis step.

For *Tenebrio molitor* beetles, Finke (2007) reported a chitin content of 13.7%, whereas our method resulted in a content of 34%. It is noteworthy that Fink averaged the chitin content from acid detergent fibre (ADF), where chitin is already hydrolyzed. Our method determines higher chitin contents, especially in the isolation of chitin from mealworms, reaching concentrations of up to 8.4%. It is important to consider that in the adult stage, chitin becomes increasingly embedded in a matrix of proteins, minerals, chitooligosaccharides and other substances (Hahn et al. 2020).

Concerning *T. molitor* exuviae, a significant disparity was observed, with Nurfikari and de Boer (2021) reporting values between 7.9% and 8.6%, while our method indicated a content of 42.8%. Hahn et al. isolated chitin from BSFL in amounts between 31% and 35%. Therefore, it is conceivable that there may be an underestimation of chitin content in Nurfikari's total hydrolysis of exuviae. These findings underscore the importance of method selection, as it can significantly impact the measured chitin contents. Importantly, none of the published methods has been tested on mixed feed.

Among commercial samples, the provided results describe the relative SD seen in the range of 1.0%–8.5%. However, in the case of dog dry feed, a significantly higher relative SD of up to 33% was noted. This discrepancy can be attributed to the intricate composition of the feed and the involved production processes. The dog dry feed consists of approximately 10 main components, like rice and DM insects, alongside nearly 20 additives including minerals and vitamins. This assortment of constituents can contribute to variations in the final product. Moreover, there is a possibility that the intensive processing method, which involves transforming the feed into pellets or kibbles through extrusion, becomes more challenging for the analytical method, resulting in a higher SD in the analyses. Additionally, the hydrothermal treatment of the feed may lead to denaturation of proteins to the point where they become insoluble or poorly soluble, potentially contributing to higher random errors within the measurements. In dog food, compared to cat food, higher proportions of plant components, such as fibres found in potatoes, may interfere with the analysis of chitin. For example, during the investigation of protein solubility in KOH, the sample is dispersed in 0.2% (w/w) KOH at room temperature. Through thermal treatment, insoluble protein complexes can form, reducing solubility. This effect should ideally be eliminated during hydrolysis in boiling 0.25 M NaOH; however, it should be considered in the case of complex and highly processed matrices, as adjustments to the hydrolysis conditions may be necessary. Essentially, high starch levels are expected to adversely affect the exchange of NaOH in the hydrolysis process, potentially leading to clogging of the filter fabric. One possible solution would be a preincubation of the sample with an amylase, similar to the amylase pretreatment used for high‐starch samples in Neutral Detergent Fibre organic matter determination.

Detailed information on variations in chitin content among different commercial samples and farming products, highlighting the importance of considering the expected chitin content and potential sources of variability in chitin analysis are compiled in Table 6.

### 4.3 Influence on Crude Protein Content

Chitin has a constant nitrogen content of 6.89 g/100 g. During Kjeldahl or Dumas nitrogen analysis, this nitrogen generally falsifies the raw protein content resulting in an overestimation. Crude protein in general is calculated from the nitrogen content of the sample multiplied by a specific factor (feed, 6.25 according to [EC] 152/2009). By applying this calculation, chitin nitrogen is rated as protein nitrogen. A correction of crude protein by reducing the total nitrogen by the chitin‐bound nitrogen offers the opportunity to generate a more useful crude protein content of feed without applying sophisticated methods such as amino acid analysis. For this correction, the chitin‐bound nitrogen determined by our new method can be subtracted from the crude nitrogen determined by the Kjeldahl method. The chitin content of the samples (Table 6) ranges from 1.8 ± 0.6 (Dog dry food C) to 10.1 ± 0.1 g/100 g DM (BSFL protein meal B). The crude protein content, estimated using the factor 6.25, varies from 21.1 (Dog dry food C) to 60.0 g/100 g DM (BSFL protein meal B).

After correcting for the chitin content, the corrected protein content ranges from 20.3 ± 0.1 (Dog dry food C) to 55.3 ± 0.1 g/100 g DM (BSFL protein meal B). The absolute overestimation of protein, calculated as the difference between the crude protein content and the corrected protein content, is highest for BSFL protein meal B with 7.8 ± 0.1 and lowest for cat dry food with 2.8 ± 0.1 g/100 g DM.

These findings emphasize the need to consider the chitin content in insect‐based samples when determining their protein content. Adjusting for the chitin content can provide a more accurate estimation of the true protein content and aid in proper nutritional evaluation and formulation. The determination of accurate protein content is crucial for ensuring the nutritional needs and optimal health of animals. Therefore, it is important to consider the correction of protein values in the presence of significant chitin contents. By applying the necessary corrections to account for chitin interference, a more reliable assessment of the actual protein content can be achieved. This ensures that the formulated diets meet the specific requirements of the animals, leading to improved performance and overall well‐being. Thus, it is recommended to incorporate the correction of protein values when evaluating commercial insect/insect‐based samples, particularly when chitin content is substantial.

Additionally, the chitin content can indirectly serve as an indicator of the percentage of insect protein during monitoring and quality control. However, it is essential to have knowledge of the raw material used for accurate assessment. By understanding the chitin content and its relationship to the insect protein content, it becomes possible to evaluate the authenticity and integrity of insect‐based products. This information is valuable for ensuring transparency in the supply chain and maintaining product quality standards.

Furthermore, the overestimation of crude protein content may result in inaccuracies in product declaration and the nutritional value of the product. This is because chitin nitrogen has a low bioavailability and does not contribute significantly to the overall nitrogen balance in monogastric animals. The correction of the protein content ensures that the product meets the required standards for animal nutrition and supports proper feeding practices. Janssen et al. (2017) developed a new N‐to‐P factor (4.76 for larvae and 5.60 for insect press cake or other extracts) calculating the protein content of insects as feed components. This value is useful for single‐feed types but is not applicable to mixed feeds. Furthermore, the global protein market uses an N‐to‐P factor of 6.25. The presented method allows this factor (6.25) to be maintained while directly assigning chitin‐bound nitrogen. This way, chitin content can be accounted for, and declared as a quality parameter in both single‐feed and mixed‐feed formulations.

## 5 Conclusions

The study provides strong evidence for the accuracy and reliability of the developed method in evaluating and controlling feed quality, particularly evident through the recovery experiments and comparison with other methods found in the literature. It demonstrates that the chitin content in the lower range has minimal impact on animal performance, eliminating the need for high‐priced special instrumentation like LC‐MS in feed assessment. Furthermore, the method supposedly corrects for the overestimation of crude protein in feed samples with chitin content above 2%. By accurately quantifying chitin, the method enables precise determination of true protein content, ensuring accurate nutritional analysis and formulation of balanced animal diets.

The developed method shows a high recovery rate ranging from 98% to 108% and excellent precision indicated by an SD below 2% within the 2%–100% chitin analytical range. These results underscore its suitability for analyzing diverse feed samples, including those with complex matrices and varying fibre and nitrogen sources. With our method, it was possible to quantify alkali‐insoluble nitrogen in several feed items. With regard to the known content of insects/insect material in these samples, we believe that this nitrogen could be attributed to chitin. Further studies will have to show how certain feed ingredients and processing parameters affect the methodology. It is important that the study highlights the limitations of estimating the protein content in insect protein feed and food solely based on nitrogen content using the commonly used factor of 6.25, leading to an overestimation of the protein content. This is particularly relevant in the context of Regulation (EU) 152/2009, as the protein content is determined by regulatory authorities according to this regulation. Furthermore, different nitrogen to CP factors exist for different sources (Janssen et al. 2017). In contrast, the developed method serves as a valuable addition for accurately assessing the chitin content and evaluating the nutritional score in feed samples.

## Author Contributions

Study conception and design were carried out by Patrick Sudwischer. Manuscript preparation was carried out by Patrick Sudwischer, Björn Krüger, Werner Sitzmann and Michael Hellwig. All authors have read and approved the final version of the manuscript.

## Ethics Statement

The authors confirm that the ethical policies of the journal, as noted on the journal's author guidelines page, have been adhered to. No ethical approval was required as this is an article in the field of nutrition analytical work, and no animal experiments were conducted.

## Conflicts of Interest

The authors declare no conflicts of interest.

## Supporting information

Supporting information.

## Data Availability

The data that support the findings of this study are available from the corresponding author upon reasonable request.
