# Supplementary material for: Chitin Analysis in Insect‐Based Feed Ingredients and Mixed Feed: Development of a Cost‐Effective and Practical Method
Source: J Anim Physiol Anim Nutr (Berl). 2025 Jan 16;109(3):854–66. doi: 10.1111/jpn.14098 (PMC12091086; doi:10.1111/jpn.14098)
Supplement: Supplementary file 1 — Supporting information. [file JPN-109-854-s001.docx]

**Supplement:**

**Table S1** Nitrogen content at alkaline deproteination kinetic study ^[a]^.

| Time | Soy bean meal HP (Control) | Soy bean meal HP + 4.2 % chitin | Insect material |
| --- | --- | --- | --- |
|  |  |  |  |
| 0 | 6.183 ± 0.107 | 6.403 ± 0.024 | 4.027 ± 0.125 |
| 1 | 3.092 ± 0.054 | 3.201 ± 0.012 | 2.014 ± 0.063 |
| 5 | 1.914 ± 0.288 | 2.421 ± 0.082 | 1.201 ± 0.023 |
| 10 | 2.332 ± 0.062 | 2.764 ± 0.149 | 1.587 ± 0.004 |
| 15 | 0.517 ± 0.003 | 0.548 ± 0.160 | 0.954 ± 0.108 |
| 20 | 0.539 ± 0.098 | 0.500 ± 0.001 | 1.008 ± 0.016 |
| 25 | 0.276 ± 0.060 | 0.352 ± 0.000 | 0.802 ± 0.006 |
| 30 | 0.123 ± 0.017 | 0.337 ± 0.002 | 0.735 ± 0.001 |
| 45 | 0.037 ± 0.001 | 0.300 ± 0.017 | 0.648 ± 0.002 |
| 60 | 0.053 ± 0.005 | 0.292 ± 0.001 | 0.648 ± 0.001 |
| 75 | 0.032 ± 0.001 | 0.273 ± 0.003 | 0.609 ± 0.000 |
| 90 | 0.044 ± 0.002 | 0.298 ± 0.001 | 0.617 ± 0.006 |

[a]All results are given in g/100 g DM. All measurements were done as double determination.

**Table S2** IR-Signal Interpretation

| Wavenumber cm^-1^ | Interpretation |
| --- | --- |
| Pure chitin |  |
| 3434 | NH 2 and -OH groups stretching vibration and intermolecular hydrogen bonding |
| 3253 | NH 2 and -OH groups stretching vibration and intermolecular hydrogen bonding |
| 3099 | C-H stretching |
| 1652 | stretching of the C=O group hydrogen bonded to N–H of the neighbouring intra-sheet chain |
| 1619 | C=O with the hydroxyl-methyl group of the next chitin residue of the same chain |
| 1552 | NH 2 bending |
| 1375 | amide II (N-H bending) and amide III (C-N stretching) |
| 1065 | Secondary alcohol |
| 1000 | Primary alcohol |
| Isolated chitin TM |  |
| 3258 | NH 2 and -OH groups stretching vibration and intermolecular hydrogen bonding |
| 3092 | C-H stretching |
| 1625 | C=O with the hydroxyl-methyl group of the next chitin residue of the same chain |
| 1542 | NH 2 bending |
| 1373 | amide II (N-H bending) and amide III (C-N stretching) |
| 1065 | Secondary alcohol |
| 1011 | Primary alcohol |

**Figure S1.** A comparison between a traditional filter fabric (left) and a FibreBag fabric (right)


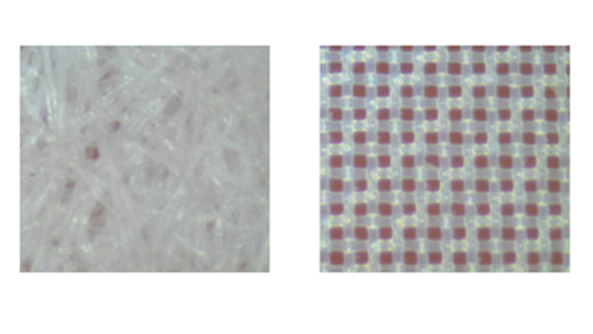


**Figure S2** IR Spectra green pure chitin, pink isolated chitin from TM.


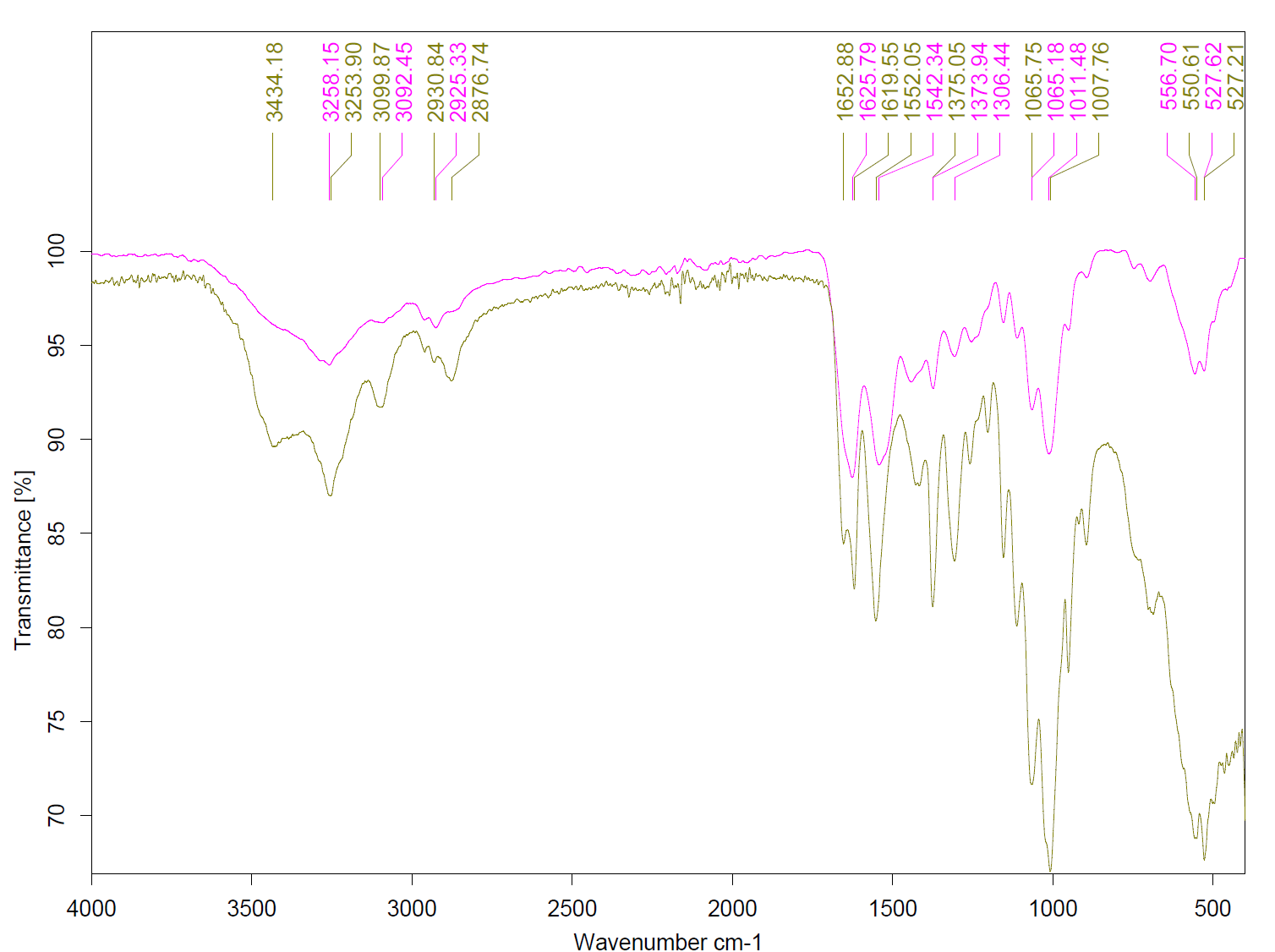


**Figure S3** Partitioning of reaction phases and linearization of the degradation rate.


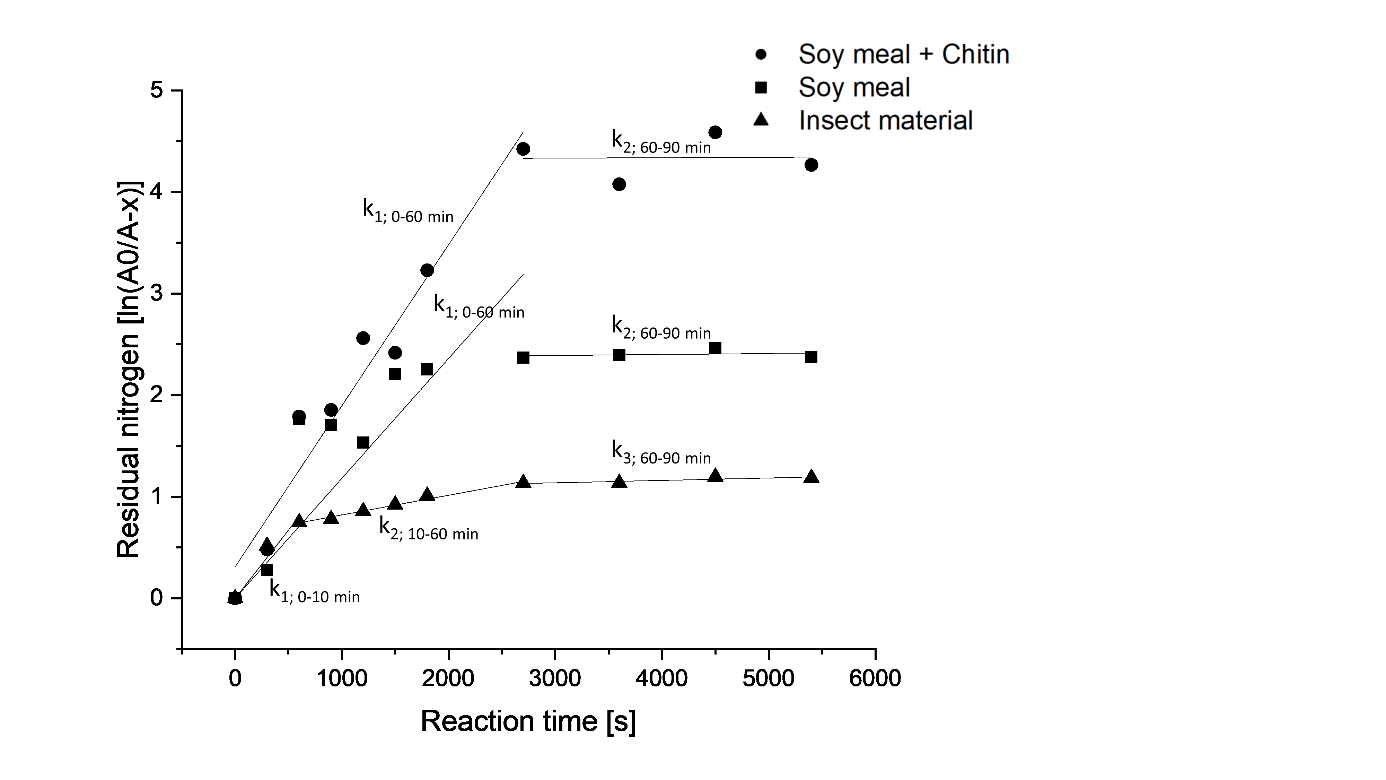


**Statistical Formulas for Method Validation**

Repeatability of measuring instruments and Repeatability:

Standard deviation in a single measurement batch (12 single sample batch)

$$s_{b}=\sqrt{\frac{1}{n-1}\sum_{i=1}^{n} \left( x_{i}-\bar{x} \right)^{2}}$$

Mean Standard Deviation

$$\bar{s_{g}}=\frac{1}{n}\sum_{b=1}^{n} s_{b}$$

In Table 5, the mean values of the standard deviation (SD) across all batches are shown, as well as the minimum and maximum values. For the repeatability of measuring a similar determination, no minimum and maximum values are determined.

Reproducibility (Between Labs)

For reproducibility and inter-laboratory comparison conditions (lab-to-lab), measurements were taken from different laboratories on various days. To achieve this, the overall standard deviation (s_t_) was determined from the standard deviations within the series (s_b_) and between the series (s_w_).

$$s_{b}=\sqrt{\frac{\sum\left( \overline{\chi}_{j}-\overline{\chi}_{ges} \right)^{2}}{n-1}}$$

$$s_{w}=\sqrt{\frac{\sum s_{j}^{2}}{n}}$$

$$s_{t}=\sqrt{s_{w}^{2}+s_{b}^{2}}$$

$$s_{j}=Standard deviation of a measureing batch$$

$$\bar{x}_{j}= Mean of the series$$

$$\bar{x}_{ges}= M\mathrm{ean}\mathrm{all}\mathrm{serie}s$$
